# Mediators of differences by parental education in weight-related outcomes in childhood and adolescence in Norway

**DOI:** 10.1038/s41598-022-09987-z

**Published:** 2022-04-05

**Authors:** Teferi Mekonnen, Anne-Lise Brantsæter, Lene F. Andersen, Nanna Lien, Onyebuchi A. Arah, Mekdes K. Gebremariam, Eleni Papadopoulou

**Affiliations:** 1 Department of Nutrition, Faculty of Medicine, Institute of Basic Medical Sciences, University of Oslo, Oslo, Norway; 2 Division for Climate and Environmental Health, Norwegian Institute of Public Health, Oslo, Norway; 3 Department of Epidemiology and Department of Statistics, University of California, Los Angeles (UCLA), Los Angeles, USA; 4 Research Unit for Epidemiology, Department of Public Health, Aarhus University, Aarhus, Denmark; 5 Department of Community Medicine and Global Health, Institute of Health and Society, Faculty of Medicine, University of Oslo, Oslo, Norway; 6 Global Health Cluster, Division of Health Service, Norwegian Institute of Public Health, Oslo, Norway

**Keywords:** Epidemiology, Paediatric research, Risk factors

## Abstract

Studies exploring mediators of socioeconomic inequalities in excess weight gain in early-life and subsequent overweight/obesity (OW/OB) among youth are limited. Thus, this study examined the mediating role of prenatal and early postnatal factors and child energy balance-related behaviours (EBRB) in the effects of parental education on (i) excess weight gain from birth to 2 years and (ii) OW/OB at 5, 8 and 14 years. The Norwegian Mother, Father and Child Cohort Study was used to include participants at the ages of 2 (n = 59,597), 5 (n = 27,134), 8 (n = 28,285) and 14 (n = 11,278) years. Causal mediation analyses using the inverse odds weighting approach were conducted. Children of low-educated parents had a higher conditional excess weight gain at 2 years compared to children of high-educated parents (total effect, RR^TE^ = 1.06; 95% CI 1.01, 1.10). The joint mediation effects of the prenatal and early postnatal factors explained most of the total effect of low education on conditional excess weight gain at 2 years. Children of low-educated parents had a higher risk of OW/OB at 5, 8 and 14 years compared to children of high-educated parents. The mediators jointly explained 63.7%, 67% and 88.9% of the total effect of parental education on OW/OB among 5, 8 and 14 year-old-children, respectively. Of the total mediated effects at 5, 8 and 14 years, the prenatal and early postnatal mediators explained 59.2%, 61.7% and 73.7%, whereas the child EBRB explained 10.3%, 15.8.0%% and 34.8%. The mediators included were found to have a considerable mediating effect in the associations explored, in particular the prenatal and early postnatal factors. If truly causal, the findings could indicate potential targets for interventions to tackle socioeconomic inequalities in OW/OB from birth to adolescence.

## Introduction

Overweight (OW) and obesity (OB) among children and adolescents represent a major public health challenge globally^1,2^. OW/OB among children and adolescents (hereafter called youth) has been associated with short and long-term health problems^3–5^. OW/OB is also known to track from childhood to adulthood^6–10^. Studies from the last decade in high-income countries indicate a plateau in the prevalence of OW/OB among youth^11–13^. However, looking into subgroups, socioeconomic position (SEP) inequalities in OW/OB among youth continue to widen^14–21^, with evidence showing that youth with low SEP have a higher burden of OW/OB or increased body mass index (BMI) than their counterparts with higher SEP.

High rates of OW/OB^7,22,23^ and SEP inequalities in BMI or OW/OB have also been documented among youth in Norway^16,24–26^. However, the reasons behind such inequalities remain poorly understood. Socioeconomic inequalities in OW/OB among youth could emerge early in life and continue throughout childhood owing to exposure to early-life determinants of OW/OB and child energy balance-related behaviours (EBRB), which vary by SEP. In this regard, prenatal factors (e.g. pre-pregnancy OW/OB, maternal diet, hypertension during pregnancy, gestational diabetes, smoking during pregnancy), and early postnatal factors (i.e. breastfeeding duration/initiation, weaning)^27,28^, and child EBRB (i.e. dietary, sedentary and physical activity behaviours)^29–32^ are among the determinants of OW/OB among youth that differ by SEP. Studies have also shown a widening social gradient in the parental lifestyle risk behaviours^33,34^, pre-pregnancy BMI/OW/OB^35^, and childhood and adolescent EBRB^1,36^. However, knowledge regarding to what extent these determinants contribute to the existing socioeconomic inequalities in OW/OB among youth is limited.

In addition, according to the Developmental Origins of Health and Diseases (DOHaD) hypothesis^37^, early-life factors including maternal weight status, maternal history of chronic diseases, smoking during pregnancy, and maternal nutrition^27,38,39^ can play a role in increasing the risk of postnatal early rapid growth and subsequent OW/OB. Studies have documented socioeconomic inequalities in early growth^40–42^. Early rapid growth has also been found to be associated with an increased risk of childhood and later life OW/OB and adverse health outcomes^43^. However, knowledge of the mediating role of the prenatal and early postnatal factors in the association between SEP and early rapid growth is limited.

Understanding whether and to what extent the above-mentioned factors explain SEP differences in early rapid growth and subsequent OW/OB among youth requires formal mediation analysis. Modern causal mediation analysis based on the potential outcomes framework and the structural causal model framework is used to investigate the mechanisms by which an exposure variable influences an outcome variable through other intermediate variables^44^. There is a limited number of studies focusing on mediators of socioeconomic inequalities in early rapid weight growth and subsequent OW/OB among youth^40,42,45^. Furthermore, most of the existing studies were based on cross-sectional data at a single age point and were based on traditional regression-based approaches that can have limitations in terms of accommodating interactions between multiple mediators. Thus, there is a need for studies with a long duration of follow-up with multiple time points and the application of recent approaches to mediation analysis. This allows for the decomposition of effects of interest within the potential outcomes framework by accommodating multiple mediators and interactions between exposure and hypothesized mediators^44,46,47^. In this regard, an inverse odds weighting (IOW) approach is a recently developed method to mediation analysis that could be used to decompose the effects of interest within the potential outcomes framework^46,47^.

Therefore, using data from a large population-based pregnancy cohort study and applying the IOW approach to mediation analysis*,* the present study aimed to examine the mediating role of the prenatal and early-postnatal factors (up to 2 years) and child EBRB in the effect of parental SEP on (i) conditional excess weight gain at 2 years and (ii) OW/OB among children and adolescents aged 5, 8 and 14 years*.*

## Methods

### Study design and sample

The Norwegian Mother, Father and Child Cohort Study (MoBa) is an ongoing population-based pregnancy cohort study conducted by the Norwegian Institute of Public Health^48^. Participants were recruited from all over Norway from 1999 to 2008. The invitation to participate was sent to pregnant women prior to the first ultrasound examination offered to all free of charge. The women consented to participation in 41% of the pregnancies. The cohort now includes 114,500 children, 95,200 mothers and 75,200 fathers. Included women were asked to respond to questionnaires received at week 17, 22 and 30 of their pregnancy, and when the child was 6 months, 18 months, 3 years, 5 years, 7 years and 8 years old. Participating children were invited to fill in questionnaires at age 13 and 14 years. The current study is based on version 12 of the quality-assured data files released for research in January 2019, with linkage to the Medical Birth Registry of Norway. The establishment of MoBa and the initial data collection was based on a license from the Norwegian Data Protection Agency and approval from The Regional Committees for Medical and Health Research Ethics. The MoBa cohort is now based on regulations related to the Norwegian Health Registry Act. The current study was approved by the Regional Committees for Medical and Health Research Ethics (REK) (REK 2018/470). Written informed consent was obtained from all mothers who consented to participate in the study. All experiments were performed in accordance with relevant guidelines and regulations.

**Figure 1:**
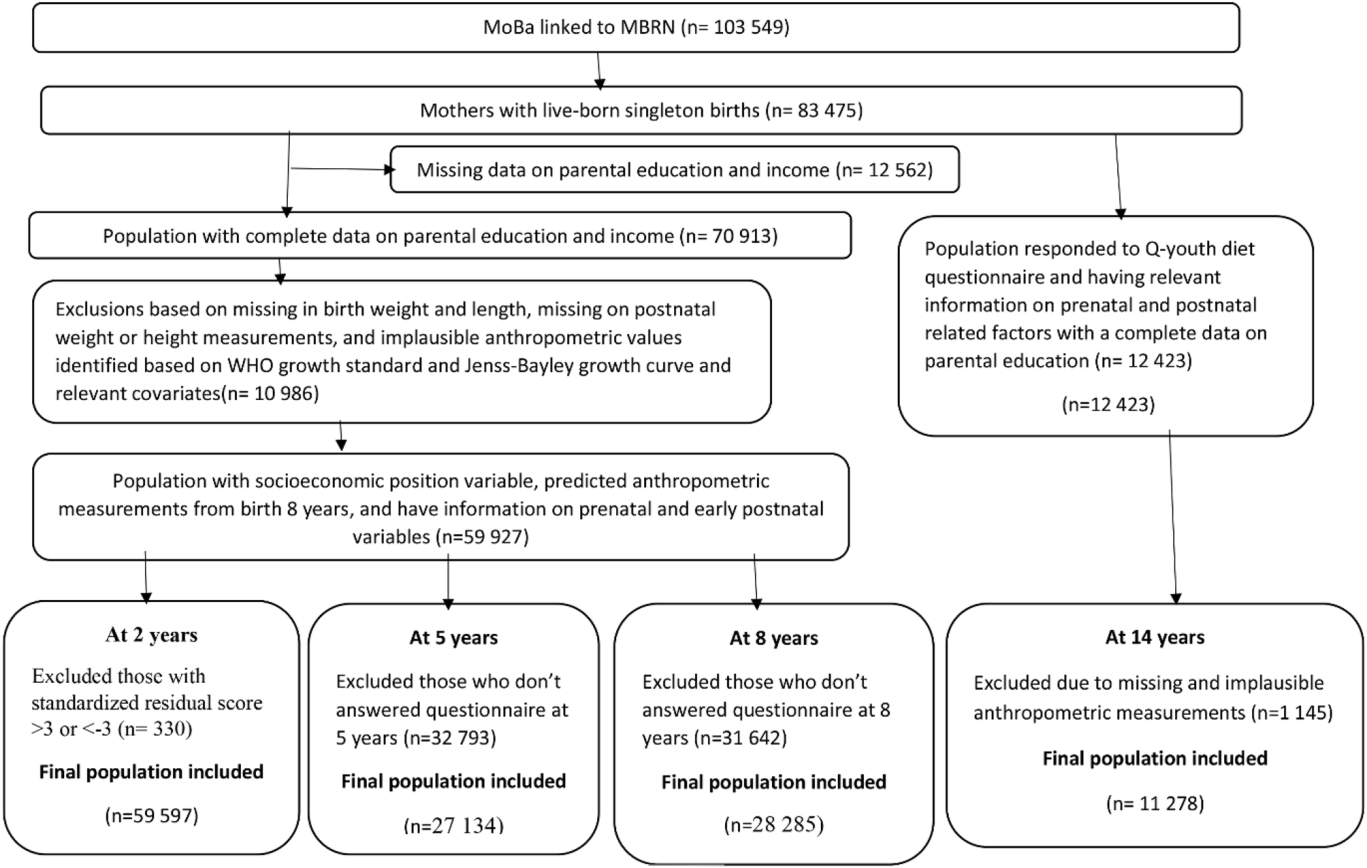
Flow chart of the study population.

After exclusion of women with multiple gestations, children with malformations and chromosomal abnormalities, 83,475 mothers with live-born singleton births remained. Of these, 70,913 had complete data on the parental level of education and income status. Further exclusions for our study population at 2, 5 and 8 years were made based on missing data on birth weight and length, missing postnatal weight or height/length measurements, implausible anthropometric values, have no relevant information on prenatal and early postnatal factors, and those who did not respond to the questionnaires at the age points considered, yielding a final sample at 2 years (n = 59,597), 5 years (n = 27,134) and 8 years (n = 28,285). For our study population at 14 years, of those with live-born singleton births, further exclusions were made to those who did not respond to the Q-youth diet questionnaire, have no relevant information on prenatal and early postnatal factors, had no complete data on the parental education and those with missing or implausible anthropometric measurements, yielding final sample at 14 years (n = 11,278) (Fig. 1).

### Measurements

Parental socioeconomic position (SEP) was measured by self-reported maternal and paternal level of education during pregnancy (baseline questionnaire at recruitment). The educational level of the parent with the longest education was used to define the parental level of education. First, parental level of education was defined into three categories as low (≤ 12 years of education), medium (13–16 years of education) and high (≥ 17 years of education). The parental level of education was then categorized into a binary variable (low education vs. high education) by merging low and medium levels of education. The “low education” represents those who completed ≤ 16 years of education, and “high education” category represents those who completed ≥ 17 years of education**.**

The anthropometric measurements at birth were obtained from the Medical Birth Registry, while measurements in infancy and up to 8 years were routinely conducted by health professionals at well-child clinics and school health services and registered in a health card from which mothers were asked to report information into MoBa questionnaires. More detail of anthropometric measurements of children from birth to 8 years is described elsewhere^16^. The anthropometric measurements for the 14 years old (average age) were obtained from the youth dietary questionnaire (Q-youth diet) developed to monitor the youths’ food intake at the age of 13–15 years.

The outcome variables (i.e. conditional excess weight gain at 2 years, OW/OB at 5 and 8 years) were based on the predicted anthropometric measurement derived using the Jenss–Bayley growth curve model from 1 month to 8 years^49^. The weight and height of the child derived from the growth model from the age of 1 month to 8 years were used to define our outcome variables; details described elsewhere^16^. The use of a growth model helps to handle the missing anthropometric measurements and overcome the loss to follow-up. Given that a high correlation between the measured and predicted anthropometric measurements was documented^16^, the predicted anthropometric measurements were used in this study.

Conditional growth based on a regression model was used to define conditional excess weight gain at two years. This model overcomes collinearity issues associated with repeated measures^50^ and has been used to define child anthropometric growth outcomes in previous studies^51–53^. The conditional weight of the child at 2 years was modelled by regressing the weight at two years on all previous weight and height measures and height at 2 years. Conditional growth at 2 years is the difference between the observed and expected weights of the child at 2 years if the individual’s growth had followed their growth trajectory predicted by the model between 18 and 24 months. Then, the children were defined as having “excess conditional weight gain at 2 years” if the standardized residual score was > 1 and “not having excess conditional weight gain at 2 years if the standardized residual score was ≤ 1. Children’s OW/OB at the age of 5, 8 and 14 years were defined based on revised International Obesity Task Force BMI cut-offs (kg/m^2^)^54^.

### Potential mediating variables

Supplementary Figure S1 shows the mediators included at different age points. The choice of potential mediators considered for our outcomes from birth to 2 years, and at 5, 8 and 14 years was based on evidence from the literature^27–32^, our research objectives, causal assumptions and data availability.

At 2 years, the prenatal and early postnatal factors included in the mediation model were maternal smoking in early pregnancy (never vs. ever smoked), maternal weight gain during pregnancy (in kg), maternal pre-pregnancy OW/OB (no vs. yes), paternal OW/OB (no vs. yes), maternal hypertension during pregnancy (yes vs.no), parity (primiparous vs. multiparous), maternal age at birth of a child (< 30 years vs. ≥ 30 years), gestational age (in weeks), breastfeeding up to 6 months (no vs. yes), and child age at introduction of solid food (< 6 months vs. ≥ 6 months) and household income (below-average income vs. above-average income, defined in accordance with data from the statistics Norway at the time of data collection (1999–2008), and defined as “below average income” families with both parents earning < 300,000 NOK per year and as “above average income” if the income of at least one parent, or both, was ≥ 300,000 NOK).

At 5 years, the prenatal and early postnatal factors (up to 2 years) included in the mediation model were birth weight, conditional excess weight gain at 2 years (yes vs. no) and all the prenatal and early postnatal factors included at 2 years. The child EBRB included in the mediation model were breakfast intake (skip vs. consume daily), sleep hours (< 8 h/day vs. ≥ 8 h/day), weekly screen time (≤ 2 h/day vs. > 2 h/day), and own TV in the bedroom (no vs. yes).

At 8 years, the prenatal and early postnatal factors (up to 2 years) included in the mediation model were birth weight, conditional excess weight gain at 2 years (yes vs. no), and prenatal and early postnatal factors included at 2 years. Summer and winter out of school physical activity (≤ 2 h/week vs. ≥ 3 h/week), child-adherence to a Mediterranean-like diet estimated using a food frequency-based Mediterranean Diet Score (fMDS) (low adherence vs. high adherence), sleep hours (≤ 8 h/day vs. ≥ 9 h/day) and weekday screen time (< 1 h/day vs. ≥ 1 h/day) were the child EBRB included into the mediation model.

At 14 years, the prenatal and early postnatal factors (up to 2 years) included in the mediation model were birth weight, conditional excess weight gain at 2 years (yes vs. no), and prenatal and early postnatal factors included at 2 years. The child EBRB included were sleep duration (< 8 h/day vs. ≥ 8 h/day), physical activity (≤ 2 h/week, 3–4 h/week, 5–7 h/week, ≥ 8 h/week), participation in organized sport (no vs.yes ), weekly screen time (≤ 2 h/day vs. > 2 h/day), fMDS (low adherence vs. high adherence) and breakfast intake (skip vs. consume daily).

### Potential confounding variables

Ethnicity was assessed based on the language spoken by mother or father defined by mother tongue other than Norwegian (yes vs. no) and maternal civil status at birth of the child (not living with a partner vs. living with a partner) were the potential confounders considered in our study.

### Statistical analysis

Stata version 16 statistical software (Stata Corporation, College Station, Texas, USA) was used to perform the data analyses.

Parental education differences in the included anthropometric outcomes, potential mediators and confounders were assessed using a chi-square test for categorical variables and an independent t-test for continuous variables.

Theoretical causal diagrams for the links between parental education and our outcome variables at different age points are shown in the supplementary file (Supplementary Fig. S1). Mediation analyses were performed using the IOW method^46^, within the potential outcomes framework, to decompose the total effect (TE) into natural direct (NDE) and natural indirect effects (NIE) without having to fit any model for the mediators. In this study, the NDE captures what the effect of parental education (high vs. low) on the outcome variables (i.e., conditional excess weight gain at 2 years, OW/OB at 5, 8 and 14 years) would remain if we were able to disable the pathway from exposure to the mediators. The NIE is the difference between the counterfactual outcomes for an exposed individual (parental education = low) with the mediators set to the value it would normally take when an individual is exposed (parental education = low) compared to the same exposed individual with the mediator set to the value it would normally take when the individual is unexposed (parental education = high). The TE captures how much the outcome would change overall for change in the exposure status from unexposed (parental education = high) to exposed (parental education = low). Multiple imputations via chained equations using 30 multiple imputed datasets were performed to impute missing data for the potential mediators. The implementation of the IOW approach to estimate TE, NDE, and NIE using multiple imputations is described by Nguyen et al. and the Stata code provided in the web appendix 4 was used for our analyses^46^.

The NDE, NIE, and TE of parental education on excess weight gain in early life and OW/OB at 5, 8, and 14 years were estimated. Two IOW models were used. The first model included all mediators (prenatal and postnatal) together. Thereafter a model that explored the independent mediating role of (i) prenatal and early postnatal factors (up to 2 years), and (ii) child energy balance-related behaviours at the age 5, 8 and 14 years was built at ages 5, 8, and 14 years. Analyses were carried out in generalized linear models with the Poisson family and log link function. Bootstrapping based on 200 replications was used to derive confidence interval (CI) for mediation parameters. The estimates were presented as relative risks (RR) with 95% CI. Finally, the proportion mediated was calculated using the formula: [RR^NDE^ (RR^NIE^ − 1)/(RR^NDE^ × RR^NIE^ − 1)] × 100.

The mediation analysis required a series of causal assumptions namely: (i) that all variables especially the exposure, mediator and outcomes are sufficiently or well defined; (ii) positivity; (iii) consistency and composition; (iv) no uncontrolled confounding of the exposure–mediator, the exposure-outcome, the mediator–outcome relations given measured covariates; (v) no exposure-induced mediator–outcome confounding; (vi) no other sources of bias; and (vii) no model misspecification^44^.

Sensitivity analyses were performed to assess: (i) how strong unmeasured confounders would have to be related to both the mediators and the outcome to substantially change our conclusions for the mediated effect using mediational E-values^55^ and (ii) if there was an association between the loss to follow-up across age points (i.e. attrition variable generated at 8 and 14 years) and OW/OB.

## Results

A total of 59,597, 27,134, 28,285 and 11,278 families were included with children at the age of 2, 5, 8 and 14 years, respectively. The percentage of children with a low parental level of education was 63.2%, 57.5%, 59.4% and 66.4% at the age of 2, 5, 8 and 14 years, respectively.

Table 1 shows the proportion of participants with conditional excess weight gain at 2 years, and with OW/OB at 5, 8 and 14 years, and the distribution of potential mediators and confounders, stratified by parental level of education. The results showed that children with low parental education were more likely to experience conditional excess weight gain at 2 years and OW/OB at 5, 8 and 14 years compared to children with high-educated parents. Similarly, those children of low parental level of education showed unfavourable outcomes for the potential mediators assessed (Table 1).

**Table 1:** Characteristics of study participants at 2 years (n = 59,597), 5 years (n = 27,134), 8 years (n = 28,285) and 14 years (n = 11,278) by parental level of education, the MoBa study.

Variables		Parental educational level	
	(%)	% Low	% High
**At 2 years**			
Conditional excess weight gain at 2 years	No (86.6)	86.3	87.1
	Yes (13.4)	13.7	12.9
Ethnicity (language spoken other than Norwegian)	No (84.4)	87.0	79.9
	Yes (15.6)	13.0	20.1
Parity	Primiparaous (46.11)	44.2	49.3
	Multiparous (53.9)	55.8	50.7
Maternal age	< 30 year-old (42.7)	49.6	30.9
	≥ 30 year-old (57.3)	50.4	69.1
Maternal civil status	Not living with a partner (2.4)	2.8	1.8
	Living with a partner (97.6)	97.2	98.2
Household income	High (71.6)	64.0	84.6
	Low (28.4)	36.0	15.4
Birth weight	Mean ± SD	3.6 ± 0.5	3.6 ± 0.5
Gestational age	Mean ± SD	39.6 ± 1.6	39.7 ± 1.5
Maternal weight gain during pregnancy	Mean ± SD	15.2 ± 6.0	14.7 ± 5.1
Maternal smoking during pregnancy	Never smoke (76.2)	70.6	85.7
	Ever smoke (23.8)	29.4	14.3
Pre-pregnancy OW/OB	Not OW/OB (68.8)	64.3	76.5
	OW/OB (31.2)	35.7	23.5
Paternal OW/OB	Not OW/OB (44.6)	41.2	50.4
	OW/OB (55.4)	58.8	49.6
Maternal hypertension during pregnancy	No (99.0)	98.9	99.2
	Yes (1.0)	1.1	0.8
Breastfeeding up to six months	No (22.7)	27.0	15.4
	Yes (77.3)	73.0	84.6
Age at introduction of solid food	< 6 months (47.7)	49.6	44.4
	≥ 6 months (52.3)	50.4	55.7
**At 5 years**			
Overweight/obesity	No (82.0)	80.5	84.2
	Yes (18.0)	19.5	15.8
Parity	Primiparaous (50.0)	47.9	52.8
	Multiparous (50.0)	52.1	47.2
Maternal age	< 30 years (40.0)	47.1	30.5
	≥ 30 years (60.0)	52.9	69.5
Household income	High (78.3)	71.5	87.5
	Low (21.7)	28.5	12.5
Maternal hypertension	No (99.1)	99.0	99.2
	Yes (0.9)	1.0	0.8
Maternal weight gain during pregnancy	Mean ± SD	14.9 ± 5.8	14.5 ± 5.0
Maternal smoking	Never smoke (80.8)	75.6	87.8
	Ever smoke (19.2)	24.4	12.2
Pre-pregnancy OW/OB	Not OW/OB (69.8)	64.6	76.8
	OW/OB (30.2)	35.4	23.2
Paternal OW/OB	Not OW/OB (45.1)	40.7	50.9
	OW/OB (54.9)	59.3	49.1
Birth weight	Mean ± SD	3.6 ± 0.5	3.6 ± 0.5
Breastfeeding up to 6 months	No (19.8)	24.4	13.6
	Yes (80.2)	75.6	86.4
Breakfast intake	Skip (4.8)	5.3	4.3
	Consume daily (95.2)	94.8	95.7
Screen time	≤ 2 h/day (84.5)	82.6	87.0
	> 2 h/day (15.5)	17.4	13.0
Sleep	≤ 8 h/day (1.0)	1.2	0.8
	> 9 h/day (99.0)	99.2	98.8
Own TV at bedroom	No (93.1)	90.5	96.6
	Yes (6.9)	9.5	3.5
Conditional excess weight gain at 2 years	No (85.9)	85.6	86.3
	Yes (14.1)	14.4	13.7
**At 8 years**			
Overweight/obesity	No (92.7)	91.5	94.5
	Yes (7.3)	8.6	5.5
Maternal age	< 30 years (39.4)	45.9	30.2
	≥ 30 years (60.5)	54.1	69.9
Parity	Primiparaous (46.7)	44.3	50.4
	Multiparous (53.2)	55.7	49.6
Household income	High (75.7)	68.5	86.3
	Low (24.3)	31.5	13.7
Maternal hypertension	No (99.1)	99.0	99.3
	Yes (0.9)	1.0	0.8
Maternal weight gain during pregnancy	Mean ± SD	15.0 ± 5.8	14.5 ± 5.0
Maternal smoking during pregnancy	Never smoke (80.2)	75.1	87.7
	Ever smoke (19.8)	24.9	12.3
Pre-pregnancy OW/OB	Not OW/OB (70.1)	65.4	77.0
	OW/OB (29.9)	34.6	23.0
Paternal OW/OB	Not OW/OB (45.3)	41.2	51.3
	OW/OB (54.7)	58.8	48.7
Birth weight	Mean ± SD	3.6 ± 0.5	3.6 ± 0.5
Breastfeeding up to 6 months	No (80.7)	23.6	13.1
	Yes (19.3)	76.5	86.9
Age at introduction of solid food	< 6 months (48.1)	50.3	44.9
	≥ 6 months (51.9)	49.7	55.1
Conditional excess weight gain at 2 years	No (85.3)	84.7	86.0
	Yes (14.8)	15.3	14.0
Summer out of school physical activity	≤ 2 h/week (11.7)	12.8	10.1
	> 3 h/week (88.3)	87.2	89.9
Winter out of school physical activity	≤ 2 h/week (16.3)	18.2	13.5
	> 3 h/week (83.7)	81.8	86.5
Screen time	< 1 h/day (49.3)	43.5	57.7
	≥ 1 h/day (50.7)	56.5	42.3
Sleep	≤ 8 h/day (1.3)	1.4	1.0
	≥ 9 h/day (98.7)	98.6	99.0
fMDS adherence	Low adherence (64.8)	66.2	62.8
	High adherence (35.3)	33.8	37.2
**At 14 years**			
Overweight/obesity	No (88.6)	87.2	90.3
	Yes (11.4)	12.8	8.7
Maternal age	< 30 years (39.7)	45.2	28.7
	≥ 30 years (60.3)	54.7	71.3
Parity	Primiparous (37.9)	37.1	39.6
	Multiparous(62.0)	62.9	60.4
Household income	High (66.0)	58.8	80.2
	Low (34.0)	41.2	19.8
Maternal hypertension	No (99.2)	99.1	99.3
	Yes (0.8)	0.9	0.7
Maternal smoking during pregnancy	Never smoke (77.3)	72.8	86.3
	Ever smoke (22.7)	27.2	13.7
Pre-pregnancy OW/OB	Not OW/OB (68.4)	65.2	74.5
	OW/OB (31.6)	34.8	25.4
Paternal OW/OB	Not OW/OB (45.6)	42.4	51.8
	OW/OB(54.4)	57.6	48.2
Maternal weight gain during pregnancy	Mean ± SD	15.4 ± 6.4	14.8 ± 5.3
Birth weight	Mean ± SD	3.6 ± 0.5	3.7 ± 0.5
Breastfeeding up to 6 months	No (19.4)	22.9	12.6
	Yes (80.6)	77.1	87.4
Conditional excess weight gain at 2 years	No (86.1)	85.9	86.5
	Yes (13.9)	14.1	13.5
Sleep	< 8 h/day (20.1)	21.9	16.6
	≥ 8 h/day (79.9)	78.1	83.4
Physical activity	≤ 2 h/week (22.0)	23.4	19.3
	3–4 h/week (27.2)	27.5	26.5
	5–7 h /week (32.1)	31.6	33.1
	≥ 8 h /week (18.7)	17.5	21.1
Participation in organized sport	No (25.6)	28.3.3	20.3
	Yes (74.4)	71.6	79.7
Screen time week-day	≤ 2 h/day (23.9)	22.2	27.2
	> 2 h/day (76.2)	77.8	72.8
Breakfast intake	Skip (26.6)	29.2	22.4
	Consume daily (74.4)	70.8	78.6
fMDS adherence	Low adherence (65.0)	67.0	61.0
	High adherence (35.0)	33.0	39.0

The sample size varies between some variables due to missing data: the potential confounders for all age points were listed at 2 years.

*fMDS* adherence to a Mediterranean-like diet estimated using a food frequency-based Mediterranean Diet Score.

### Mediation analyses results

The estimated natural direct, natural indirect and total effects of parental education on children’s conditional excess weight gain at 2 years, and OW/OB at 5, 8 and 14 years are shown in Table 2.

**Table 2:** Total, direct and indirect effects of parental education on early excess weight gain and OW/OB at 5, 8 and 14 years of the youth in the MoBa-study.

Parental educational level	2 years (n = 59,597)		5 years (n = 27,134)		8 years (n = 28,285)		14 years (n = 11 278)	
	Conditional excess weight gain at 2 years		Overweight/obesity		Overweight/obesity		Overweight/obesity	
	RR	95% CI	RR	95% CI	RR	95% CI	RR	95% CI
Total effect	1.06	1.01–1.10	1.23	1.16–1.30	1.55	1.40–1.72	1.45	1.28–1.64
Natural direct effect	0.97	0.92–1.01	1.08	1.03–1.15	1.18	1.06–1.32	1.05	0.93–1.19
Natural indirect effect	1.09	1.07–1.18	1.13	1.09–1.17	1.31	1.24–1.39	1.38	1.29–1.47
Proportion mediated (%)	–	–	63.7	56.6–75.6	67.0	61.7–80.9	88.9	74.6–135.1

Mediation model at 2 years adjusted for ethnicity and maternal civil status; Mediation models at 5, 8 and 14 years adjusted for ethnicity and maternal civil status.

Children of low educated parents had a higher risk of conditional excess weight gain at 2 years compared to their peers with high-educated parents (total effect, RR^TE^ = 1.06). The prenatal and early postnatal mediators included at 2 years explained most of the total effect of parental education on conditional excess weight gain at 2 years (natural indirect effect, RR^NIE^ = 1.09). Children of low educated parents had a higher risk of OW/OB at 5 years (RR^TE^ = 1.23), 8 years (RR^TE^ = 1.55) and 14 years (RR^TE^ = 1.45) compared to their counterparts with high-educated parents. Of the total effect of parental education on OW/OB among 5-year-olds, 63.7% was explained by the joint effects of mediators included at 5 years (RR^NIE^ = 1.13). Similarly, 67% of the total effect of parental education on OW/OB among 8-year-olds was explained by the joint effects of mediators included at 8 years (RR^NIE^ = 1.31). The natural direct effect of education on OW/OB at 5 and 8 years remained non-null (RR^NDE^ = 1.08 and RR^NDE^ = 1.18, respectively). Among 14-year-old adolescents, the joint effect of mediators included at 14 years explained 88.9% of the total effect of parental education on OW/OB (RR^NIE^ = 1.38).

Further analyses showed that most of the effect of parental education on OW/OB at the age of 5, 8 and 14 years was explained by the prenatal and early postnatal factors (up to 2 years), explaining 59.2% of the TE at 5-years (RR^NIE^ = 1.13), 61.7% of the TE at 8-years (RR^NIE^ = 1.28) and 73.7% of the TE at 14-years (RR^NIE^ = 1.30). Child EBRB at 5, 8 and 14 years explained 10.3% (RR^NIE^ = 1.02), 15.8% (RR^NIE^ = 1.06) and 34.8% (RR^NIE^ = 1.12) of the total effect of parental education on OW/OB among 5, 8 and 14 year-old children, respectively (Table 3).

**Table 3:** Total, direct and indirect effects of parental education on OW/OB at ages 5, 8 and 14 years, in the MoBa study.

Parental educational level	5 years (n = 27,134)		8 years (n = 28,285)		14 years (n = 11 278,	
	Overweight/obesity		Overweight/obesity		Overweight/obesity	
	RR	95% CI	RR	95% CI	RR	95% CI
**Mediators included: prenatal and early postnatal factors (up to 2 years)**						
Total effect	1.23	1.17–1.29	1.55	1.41–1.70	1.45	1.28–1.64
Natural direct effect	1.09	1.04–1.15	1.21	1.10–1.33	1.12	0.98–1.27
Natural indirect effect	1.12	1.09–1.16	1.28	1.22–1.35	1.30	1.22–1.38
Proportion mediated (%)	59.2	55.1– 70.1	61.7	57.5–70.8	73.7	64.1–110.2
**Mediators included: child health-related behaviours**						
Total effect	1.23	1.17–1.29	1.55	1.42–1.69	1.45	1.29–1.63
Natural direct effect	1.21	1.14–1.28	1.47	1.35–1.60	1.29	1.15–1.46
Natural indirect effect	1.02	1.01–1.03	1.06	1.04–1.07	1.12	1.09–1.15
Proportion mediated (%)	10.3	7.5–12.1	15.8	13.4–15.7	34.8	32.3–40.8

All models were adjusted for ethnicity and maternal civil status.

### Sensitivity analyses

With an observed natural indirect effect risk ratio of 1.09, an unmeasured confounder that was associated with both the mediators and conditional excess weight gain at 2 years by risk ratios of 1.40-fold each, conditional on the measured confounders, could explain away the estimate but weaker joint unmeasured confounder associations could not.

With an observed natural indirect effect risk ratio of 1.13 at 5 years, 1.31 at 8 years and 1.38 at 14 years, an unmeasured confounder that was associated with both the mediators and OW/OB by risk ratios of 1.51-fold each at 5 years, 1.95-fold each at 8 years and 2.10-fold each at 14 years, conditional on the measured confounders, could explain away the estimates but weaker joint unmeasured confounder associations could not.

Sensitivity analysis assessing the association between the loss to follow-up across age points and our outcome variable showed no substantive association between attrition (generated at 8 years and OW/OB at 14 years [OR 1.08, 95% CI 0.97, 1.20] (data not shown).

## Discussion

This study aimed to quantify the mediation effect of prenatal and early postnatal factors (up to 2 years) and child energy balance-related behaviours in the association between parental level of education and weight-related outcomes among 2, 5, 8 and 14-year-old children in the MoBa study using the IOW approach. Through the IOW, a recently developed method for mediation analysis, we were able to decompose the total effect of parental education on children’s weight-related outcomes into natural direct and natural indirect effects. IOW approach to mediation analysis is a method of quantifying the joint mediation of the exposure effect by a set of mediators. This method has advantages over the other traditional-regression methods by accommodating multiple mediators regardless of their scales and avoids building a model for each mediator irrespective of its scale, which is computationally prohibitive as the number of mediators increases^46,47^. In addition*,* the traditional regression-based approaches to mediation analysis presuppose no exposure–mediator or mediator–mediator interactions. However, holding these assumptions seems difficult in our context because OW/OB is attributable to a complex network of lifestyle factors known to interact with each other. There is evidence showing that ignoring interactions can potentially introduce biased conclusions^44^. Our study decomposed the effect of parental education on weight-related outcomes from birth to adolescence by utilizing a causal mediation analysis approach that accommodates multiple-mediators and could provide a valid estimate regardless of whether interactions between mediators, between mediator(s) and exposures or between mediator(s) and confounding variables are present without having to assess and specify them.

We demonstrated that most of the total effect of parental education on children’s conditional excess weight gain at 2 years, and nearly 64 to 89% of the total effect of parental education on OW/OB among 5, 8 and 14-year-olds were explained by the joint effect of prenatal and early postnatal factors (up to 2 years) and child energy balance-related behaviours included at each age points assessed.

It is important to understand the mechanisms explaining socioeconomic inequalities in excess weight gain in early life and inform public health interventions, given that those children who experienced early rapid growth have a higher likelihood of developing childhood and later life OW/OB and adverse health outcomes^43^. A few studies have explored the mediators explaining socioeconomic inequalities in early rapid growth using the traditional regression-based methods to mediation analysis^40,42^ and found that maternal smoking, maternal BMI, infant birth weight, gestational age at birth, maternal age and child-feeding practices mediated socioeconomic inequalities in early rapid growth. These studies have limitations in controlling for interactions between exposure and hypothesized mediators given that they have used traditional regression-based mediation analyses. Our study is the first study with a large sample size, which accounts for these limitations and indicates a substantial joint mediation effect of the prenatal and early postnatal factors for the association between parental level of education and conditional excess weight gain at 2 years.

Our findings showed that most of the total effect of parental level of education on OW/OB among 5 and 8 years old children was explained by the joint mediation effect of the prenatal and early postnatal factors (up to 2 years), and child energy balance-related behaviours identified at 5 years (explaining 64%) and 8 years (explaining 67%).

We found that nearly 90% of the total effect of parental education on OW/OB among adolescents was accounted through the joint mediation effects of prenatal and early postnatal factors (up to 2 years), and the adolescent EBRB. Mediators which we considered in our study (i.e. parental BMI, parental smoking behaviours, and child physical activity) were also reported as mediators explaining SEP differences in OW/OB among adolescents aged 11–16 years in studies from Finland and Germany^56,57^. Nevertheless, we quantified the joint mediation effect of multiple early-life factors (i.e. birth weight, maternal age, parity, household income, breastfeeding, age at introduction of solid food, and weight gain during early life), and adolescent EBRB (i.e. participation in organized sport, level of physical activity, breakfast intake, sleep hours, dietary adherence and screen time) including those mediators documented in the previous studies. To our knowledge, this is the first study that explored the joint mediation effect of the adolescent EBRB and the prenatal and early postnatal factors using a large sample size with multiple time point data.

Tackling socioeconomic inequalities in OW/OB in childhood and later in life requires the understanding of critical periods for the emergence of socioeconomic inequalities in OW/OB. In this regard, it is evident that critical periods for socioeconomic inequalities in OW/OB could begin early in life^58,59^ through exposure to prenatal and early postnatal factors that vary by SEP^27^. Our result showed a substantial contribution of the prenatal and early postnatal factors (up to 2 years) taken together in explaining the existing socioeconomic inequalities in OW/OB across childhood and adolescence. Public health efforts aimed at tackling socioeconomic differences in childhood and later life OW/OB need to target the identified modifiable early-life factors of OW/OB. The DOHaD hypothesis indicates that exposure to environmental factors during the critical window development period might predispose to diseases in later-life^37^. In this regard, the contribution of exposure to early life factors to the subsequent development of childhood overweight/obesity has been reported by previous longitudinal studies with evidence demonstrating a higher risk of being overweight/obese in early childhood, mid-childhood, and early adolescence among those exposed to early life factors^60–63^. The substantial contribution of early-life factors to the inequalities documented in our study, and the finding of other studies^60–63^ may support the DOHaD hypothesis where the fetal and early-life factors influence the development of subsequent overweight/obesity. Thus, the prenatal period could be one of the critical periods to be targeted for public health efforts to early OW/OB prevention^63^. In this regard, our results showed the substantial contribution of the prenatal factors (i.e. weight gain during pregnancy, maternal OW/OB, paternal OW/OB, maternal smoking during pregnancy, maternal age at pregnancy, parity, household income) in explaining the existing socioeconomic inequalities in weight-related outcomes among children and adolescent, which showed a higher percentage mediated effect than early postnatal factors (Supplementary Table S1).

We found that the mediating role of the EBRB from preschool to adolescence was relatively small compared to the prenatal and early postnatal factors (up to 2 years). The relatively small proportion of the mediated effect of the energy balance-related behaviours at these ages might be due to the measurement approaches used to assess these variables. Most of the child EBRB including, physical activity and sedentary behaviours included in this study were based on data reported by parents (5 and 8 years) and children (14 years). Studies have shown that indirect measures (i.e. parental-report or self-report) of health-related behaviours have limitations, and tend to overestimate physical activity behaviours^64–66^ and underestimate sedentary behaviours^65^. The use of self-reported or parent-reported data could introduce information bias in the NIE if the mediators suffered from differential or dependent measurement error possibly affected or differential by SEP. Thus, using indirectly measured variables as a mediator in our analyses may not show the exact contributions of child energy balance-related-behaviours in explaining the existing socioeconomic inequalities in OW/OB among 5, 8 and 14-year-old children. Our study result also showed that 36.3%, 33.7%, and 11.1% of the total effect of parental education on childhood OW/OB at 5, 8, and 14 years was not explained by the included mediators. Therefore, more studies exploring more mediators, including the objectively measured energy-balance related behaviours, are important to identify mediators explaining the association between SEP and child weight-related outcomes at different age points.

In this study, both modifiable and non-modifiable factors explaining SEP inequalities in child weight-related outcomes were considered. Our results of the proportion mediated with and without the non-modifiable factors indicated that there was only a small increase in the proportion mediated after accounting for the non-modifiable factors, including maternal age, parity and household income. Thus, the modifiable factors drove most of the socioeconomic inequalities in child weight-related outcomes.

There is some documented evidence of gender-specific mediation of socioeconomic position differences in adiposity among youth^67,68^. Quantifying such gender-or sex-specific effects was beyond the scope of this study. We hope that future studies would investigate how gender or sex might modify pathways explaining SEP differences in weight-related outcomes among youth.

### Policy implications

A substantial contribution of early life factors (up to 2 years) in explaining SEP differences in child weight-related outcomes from birth to adolescence indicates the need to introduce health promotion efforts targeting women of reproductive age and pregnant women towards prevention of pre-pregnancy overweight/obesity, proper weight gain during pregnancy, prevention of hypertension during pregnancy, smoking cessation, promotion of breastfeeding up to 6 months and timely introduction of solid food to combat the existing socioeconomic inequalities in child weight-related outcomes from birth to adolescence. This could be done by integrating with antenatal care follow-ups. In this regard, studies from Norway indicated an equitable distribution in the utilization of health services that are provided free of charge^69^ and a narrowing in the inequalities in the utilization of obstetric services from 1967 to 2009^70^. However, socieoeconomic inequalities in the indicators of prenatal maternal health (e.g. preterm birth, low birth weight, small for gestational age, stillbirth)^71^ and infant feeding prectices^72^ was reported. Thus, integrating interventions targeting the above mentioned early life risk factors of social inequalities in child weight-related outcomes with antenatal care follow-ups that are provided free of charge in Norway may help to reduce the observed social inequalities in child weight-related outcomes in childhood and adolescence in Norway. Furthermore, targeting early childhood for OW/OB prevention is recommended to reduce childhood and later-life OW/OB^73^. Thus, it is important to target children from preschool to adolescence to promote healthy energy balance-related behaviours.

### Strengths and limitations

The strengths of this study include the large sample size and the application of an IORW approach, a method that allows for the decomposition of effects of interest within the potential outcomes framework and accommodating multiple mediators. Furthermore, missing data for anthropometric measurements (up to 8 years) were handled with the use of a growth model and missing data for the mediators and confounders were handled by using multiple imputations.

However, this study also has weaknesses. The anthropometric measurements of the children were based on parent-reported measurements (although consultation of the children’s health card was suggested for several age points), which might introduce reporting errors. There are studies indicating reverse causality for the associations between child growth outcomes and early child feeding practices^74,75^. Therefore, the mediators: breastfeeding and age at introduction of solid food, which were considered for conditional excess weight gain at 2 years in this study, could suffer from the reverse causality that could introduce bias in our estimates. Thus, the use of mediators suffering from reverse causality for our outcomes from birth to 2 years may bias our natural indirect effect estimates, although this bias may not be substantial given that the model estimates the joint effects of mediators, including those suffering from reverse causality. Information on the separate contribution of each mediator may sometimes be necessary for intervention or policy prioritization; in this regard, the mediation analysis approach used in this study does not provide such insights into such separate effects. Nonetheless, it is often extremely difficult to disentangle such separate contributions given their complex interconnections without making unrealistic simplifying assumptions about them. Furthermore, self-selection in MoBa is also a concern. Women participating in MoBa are older, better educated and include fewer smokers than the general population of pregnant women^76^. Therefore, the mediation parameters for the associations between SEP and rapid growth from birth to 2 years, and OW/OB at 5, 8, and 14 years in the current study are likely to be underestimated; the generally small risk ratio in the mediation parameters should thus be seen in the light of this potential underestimation. Finally, another limitation in this study is that the measurements of some of the mediators, such as physical activity and sedentary behaviours, were based on self-report measures. The use of self-reported data could introduce information bias in the NIE if the mediators suffered from differential or dependent measurement error possibly affected or differential by SEP. In addition, the information collected for the mediating variables, particularly the child energy balance-related behaviours, was based on categorical responses, which may lead to loss of information and introduce misclassification bias that may underestimate the indirect effects^77^. Therefore, the results in our mediation analyses particularly, the mediating role of child energy balance-related factors, should be seen in the light of these limitations. Future work should use external data or information to conduct extensive quantitative bias analysis addressing measurement error in such a multiple-mediator setting as ours.

## Conclusion

The mediators included were found to have a considerable mediating effect in the associations explored, in particular the prenatal and early postnatal factors. If truly causal, the findings could indicate potential targets for interventions to tackle socioeconomic inequalities in OW/OB from birth to adolescence.

## Supplementary Information

Supplementary Information.

## Data Availability

The datasets analyzed during the current study are not publicly available due to data protection regulations. Researchers who want access to data sets for replication should submit an application to datatilgang@fhi.no. Access to data sets requires approval from The Regional Committee for Medical and Health Research Ethics in Norway and an agreement with MoBa.

## References

[1] World Health Organization (2017). Adolescent Obesity and Related Behaviours: Trends and Inequalities in the WHO European Region, 2002–2014.

[2] Garrido-Miguel M (2019). Prevalence and trends of overweight and obesity in European children from 1999 to 2016: A systematic review and meta-analysis. JAMA Pediatr..

[3] Di Angelantonio E (2016). Body-mass index and all-cause mortality: Individual-participant-data meta-analysis of 239 prospective studies in four continents. The Lancet.

[4] Collaborators GO (2017). Health effects of overweight and obesity in 195 countries over 25 years. N. Engl. J. Med..

[5] Sharma V (2019). A systematic review and meta-analysis estimating the population prevalence of comorbidities in children and adolescents aged 5 to 18 years. Obes. Rev..

[6] Singh AS, Mulder C, Twisk JW, Van Mechelen W, Chinapaw MJ (2008). Tracking of childhood overweight into adulthood: A systematic review of the literature. Obes. Rev..

[7] Evensen E, Wilsgaard T, Furberg A-S, Skeie G (2016). Tracking of overweight and obesity from early childhood to adolescence in a population-based cohort—The Tromsø study, fit futures. BMC Pediatr..

[8] Norris T, Bann D, Hardy R, Johnson W (2020). Socioeconomic inequalities in childhood-to-adulthood BMI tracking in three British birth cohorts. Int. J. Obes..

[9] Kristiansen AL (2015). Tracking of body size from birth to 7 years of age and factors associated with maintenance of a high body size from birth to 7 years of age—The Norwegian Mother and Child Cohort study (MoBa). Public Health Nutr..

[10] Geserick M (2018). Acceleration of BMI in early childhood and risk of sustained obesity. N. Engl. J. Med..

[11] Wabitsch M, Moss A, Kromeyer-Hauschild K (2014). Unexpected plateauing of childhood obesity rates in developed countries. BMC Med..

[12] Olds T (2011). Evidence that the prevalence of childhood overweight is plateauing: Data from nine countries. Int. J. Pediatr. Obes..

[13] Rokholm B, Baker JL, Sørensen TIA (2010). The levelling off of the obesity epidemic since the year 1999—A review of evidence and perspectives. Obes. Rev..

[14] Chung A (2016). Trends in child and adolescent obesity prevalence in economically advanced countries according to socioeconomic position: A systematic review. Obes. Rev..

[15] Bann D, Johnson W, Li L, Kuh D, Hardy R (2018). Socioeconomic inequalities in childhood and adolescent body-mass index, weight, and height from 1953 to 2015: An analysis of four longitudinal, observational, British birth cohort studies. Lancet Public Health.

[16] Mekonnen T (2021). Socioeconomic inequalities in children’s weight, height and BMI trajectories in Norway. Sci. Rep..

[17] Bouthoorn SH (2014). Development of socioeconomic inequalities in obesity among Dutch pre-school and school-aged children. Obesity.

[18] Ballon M (2018). Socioeconomic inequalities in weight, height and body mass index from birth to 5 years. Int. J. Obes..

[19] Howe LD (2011). Socioeconomic disparities in trajectories of adiposity across childhood. Int. J. Pediatr. Obes..

[20] Morgen CS (2017). Socioeconomic disparities in birth weight and body mass index during infancy through age 7 years: A study within the Danish National Birth Cohort. BMJ Open.

[21] Mekonnen T (2021). Socioeconomic inequalities in children’s weight, height and BMI trajectories in Norway. Sci. Rep..

[22] Júlíusson PB (2010). Overweight and obesity in Norwegian children: Prevalence and socio-demographic risk factors. Acta Paediatr..

[23] Glavin K (2014). Important periods of weight development in childhood: A population-based longitudinal study. BMC Public Health.

[24] Bjelland M (2010). Overweight and waist circumference among Norwegian 11-year-olds and associations with reported parental overweight and waist circumference: The HEIA study. Scand. J. Public Health.

[25] Biehl A (2013). Adiposity among children in Norway by urbanity and maternal education: A nationally representative study. BMC Public Health.

[26] Lien N, Kumar BN, Holmboe-Ottesen G, Klepp K-I, Wandel M (2007). Assessing social differences in overweight among 15-to 16-year-old ethnic Norwegians from Oslo by register data and adolescent self-reported measures of socio-economic status. Int. J. Obes..

[27] Cameron AJ (2015). A review of the relationship between socioeconomic position and the early-life predictors of obesity. Curr. Obes. Rep..

[28] Silva L (2008). Maternal educational level and risk of gestational hypertension: The generation R study. J. Hum. Hypertens..

[29] Nilsen SM, Krokstad S, Holmen TL, Westin S (2010). Adolescents’ health-related dietary patterns by parental socio-economic position: The Nord-Trøndelag Health Study (HUNT). Eur. J. Pub. Health.

[30] Mullie P, Clarys P, Hulens M, Vansant G (2010). Dietary patterns and socioeconomic position. Eur. J. Clin. Nutr..

[31] Mielke GI, Brown WJ, Nunes BP, Silva IC, Hallal PC (2017). Socioeconomic correlates of sedentary behavior in adolescents: Systematic review and meta-analysis. Sports Med..

[32] O’Donoghue G (2018). Socio-economic determinants of physical activity across the life course: A “determinants of diet and physical activity”(DEDIPAC) umbrella literature review. PLoS ONE.

[33] Ding D, Do A, Schmidt H-M, Bauman AE (2015). A widening gap? Changes in multiple lifestyle risk behaviours by socioeconomic status in New South Wales, Australia, 2002–2012. PLoS ONE.

[34] Buck D, Frosini F (2012). Clustering of Unhealthy Behaviours Over Time.

[35] Lundberg CE, Ryd M, Adiels M, Rosengren A, Björck L (2021). Social inequalities and trends in pre-pregnancy body mass index in Swedish women. Sci. Rep..

[36] Lioret S (2020). Lifestyle patterns begin in early childhood, persist and are socioeconomically patterned, confirming the importance of early life interventions. Nutrients.

[37] Gillman MW (2005). Developmental origins of health and disease. N. Engl. J. Med..

[38] Halfon N, Forrest CB, Lerner RM, Faustman EM (2018). Handbook of Life Course Health Development.

[39] Huang JS, Lee TA, Lu MC (2007). Prenatal programming of childhood overweight and obesity. Matern. Child Health J..

[40] Wang L (2018). Relationship between socioeconomic status and weight gain during infancy: The BeeBOFT study. PLoS ONE.

[41] Wijlaars LP, Johnson L, van Jaarsveld CH, Wardle J (2011). Socioeconomic status and weight gain in early infancy. Int. J. Obes..

[42] Van Den Berg G, Van Eijsden M, Galindo-Garre F, Vrijkotte T, Gemke R (2013). Low maternal education is associated with increased growth velocity in the first year of life and in early childhood: The ABCD study. Eur. J. Pediatr..

[43] Zheng M (2018). Rapid weight gain during infancy and subsequent adiposity: A systematic review and meta-analysis of evidence. Obes. Rev..

[44] VanderWeele T (2015). Explanation in Causal Inference: Methods for Mediation and Interaction.

[45] Gebremariam M, Lien N, Nianogo R, Arah O (2017). Mediators of socioeconomic differences in adiposity among youth: A systematic review. Obes. Rev..

[46] Nguyen QC, Osypuk TL, Schmidt NM, Glymour MM, Tchetgen EJT (2015). Practical guidance for conducting mediation analysis with multiple mediators using inverse odds ratio weighting. Am. J. Epidemiol..

[47] Tchetgen Tchetgen EJ (2013). Inverse odds ratio-weighted estimation for causal mediation analysis. Stat. Med..

[48] Magnus P (2016). Cohort profile update: The Norwegian mother and child cohort study (MoBa). Int. J. Epidemiol..

[49] Jenss RM, Bayley N (1937). A mathematical method for studying the growth of a child. Hum. Biol..

[50] Tu Y-K, Tilling K, Sterne JA, Gilthorpe MS (2013). A critical evaluation of statistical approaches to examining the role of growth trajectories in the developmental origins of health and disease. Int. J. Epidemiol..

[51] Horta BL (2017). Associations of linear growth and relative weight gain in early life with human capital at 30 years of age. J. Pediatr..

[52] De Franca GA (2016). Associations of birth weight, linear growth and relative weight gain throughout life with abdominal fat depots in adulthood: The 1982 Pelotas (Brazil) birth cohort study. Int. J. Obes..

[53] De Beer M (2015). Associations of infant feeding and timing of linear growth and relative weight gain during early life with childhood body composition. Int. J. Obes..

[54] Cole TJ, Lobstein T (2012). Extended international (IOTF) body mass index cut-offs for thinness, overweight and obesity. Pediatr. Obes..

[55] Smith LH, VanderWeele TJ (2019). Mediational E-values: Approximate sensitivity analysis for unmeasured mediator–outcome confounding. Epidemiology (Cambridge).

[56] Parikka S (2015). Associations between parental BMI, socioeconomic factors, family structure and overweight in Finnish children: A path model approach. BMC Public Health.

[57] Gätjens I, Hasler M, di Giuseppe R, Bosy-Westphal A, Plachta-Danielzik S (2020). Family and lifestyle factors mediate the relationship between socioeconomic status and fat mass in children and adolescents. Obes. Facts.

[58] Hanson MA, Gluckman P (2014). Early developmental conditioning of later health and disease: Physiology or pathophysiology?. Physiol. Rev..

[59] Brisbois TD, Farmer AP, McCargar LJ (2012). Early markers of adult obesity: A review. Obes. Rev..

[60] Li C, Goran MI, Kaur H, Nollen N, Ahluwalia JS (2007). Developmental trajectories of overweight during childhood: Role of early life factors. Obesity.

[61] Gillman MW (2008). Developmental origins of childhood overweight: Potential public health impact. Obesity.

[62] Robinson SM (2015). Modifiable early-life risk factors for childhood adiposity and overweight: An analysis of their combined impact and potential for prevention. Am. J. Clin. Nutr..

[63] Gillman MW, Ludwig DS (2013). How early should obesity prevention start?. N. Engl. J. Med..

[64] Adamo KB, Prince SA, Tricco AC, Connor-Gorber S, Tremblay M (2009). A comparison of indirect versus direct measures for assessing physical activity in the pediatric population: A systematic review. Int. J. Pediatr. Obes..

[65] Ayala-Guzmán CI, Ramos-Ibáñez N, Ortiz-Hernández L (2017). Accelerometry does not match with self-reported physical activity and sedentary behaviors in Mexican children. Boletín Méd. Del Hosp. Infantil de Méx. (English Ed.).

[66] Colley RC, Butler G, Garriguet D, Prince SA, Roberts KC (2019). Comparison of self-reported and accelerometer-measured physical activity among Canadian youth. Health Rep..

[67] Dollman J, Ridley K, Magarey A, Martin M, Hemphill E (2007). Dietary intake, physical activity and TV viewing as mediators of the association of socioeconomic status with body composition: A cross-sectional analysis of Australian youth. Int. J. Obes..

[68] Gebremariam MK (2019). Gender-specific mediators of the association between parental education and adiposity among adolescents: The HEIA study. Sci. Rep..

[69] Vikum E, Krokstad S, Westin S (2012). Socioeconomic inequalities in health care utilisation in Norway: The population-based HUNT3 survey. Int. J. Equity Health.

[70] Eriksen HS, Høy S, Irgens LM, Rasmussen S, Haug K (2020). Social inequalities in the provision of obstetric services in Norway 1967–2009: A population-based cohort study. Eur. J. Pub. Health.

[71] Mc Dowell, A. M. *Inequalities in Health Outcomes Among Users of Prenatal Care.* Masters Thesis thesis, University of Oslo (2020).

[72] Gimse, G. M. M. *Social inequalities and differences in social characteristics in infant feeding practices among Norwegian 6 months old infants-Spedkost 2006* Masters Thesis thesis, University of Oslo (2009).

[73] Deal BJ, Huffman MD, Binns H, Stone NJ (2020). Perspective: Childhood obesity requires new strategies for prevention. Adv. Nutr..

[74] Kramer MS, Moodie EE, Dahhou M, Platt RW (2011). Breastfeeding and infant size: Evidence of reverse causality. Am. J. Epidemiol..

[75] Marquis GS, Habicht J-P, Lanata CF, Black RE, Rasmussen KM (1997). Association of breastfeeding and stunting in Peruvian toddlers: An example of reverse causality. Int. J. Epidemiol..

[76] Nilsen RM (2009). Self-selection and bias in a large prospective pregnancy cohort in Norway. Paediatr. Perinat. Epidemiol..

[77] Blakely T, McKenzie S, Carter K (2013). Misclassification of the mediator matters when estimating indirect effects. J. Epidemiol. Community Health.

