# Supplementary material for: Mediators of differences by parental education in weight-related outcomes in childhood and adolescence in Norway
Source: Sci Rep. 2022 Apr 5;12:5671. doi: 10.1038/s41598-022-09987-z (PMC8983661; doi:10.1038/s41598-022-09987-z)
Supplement: Supplementary file 1 — Supplementary Information. [file 41598_2022_9987_MOESM1_ESM.pdf]

Mediators of differences by parental education in weight-related outcomes in childhood and adolescence in Norway

Teferi Mekonnen[1], Anne-Lise Brantsæter[2], Lene F. Andersen[1], Nanna Lien[1], Onyebuchi A. Arah[3], Mekdes K Gebremariam[4]*, Eleni Papadopoulou[5]*

[1]Department of Nutrition, Institute of Basic Medical Sciences, Faculty of Medicine, University of Oslo, Norway

[2]Division for climate and Environmental Health, Norwegian Institute of Public Health, Norway

[3]Department of Epidemiology and Department of Statistics, University of California, Los Angeles (UCLA), USA; Research Unit for Epidemiology, Department of Public Health, Aarhus University, Aarhus, Denmark.

[4]Department of Community Medicine and Global Health, Institute of Health and Society, Faculty of Medicine, University of Oslo, Norway

[5]Global Health Cluster, Division of Health Service, Norwegian Institute of Public Health, Norway

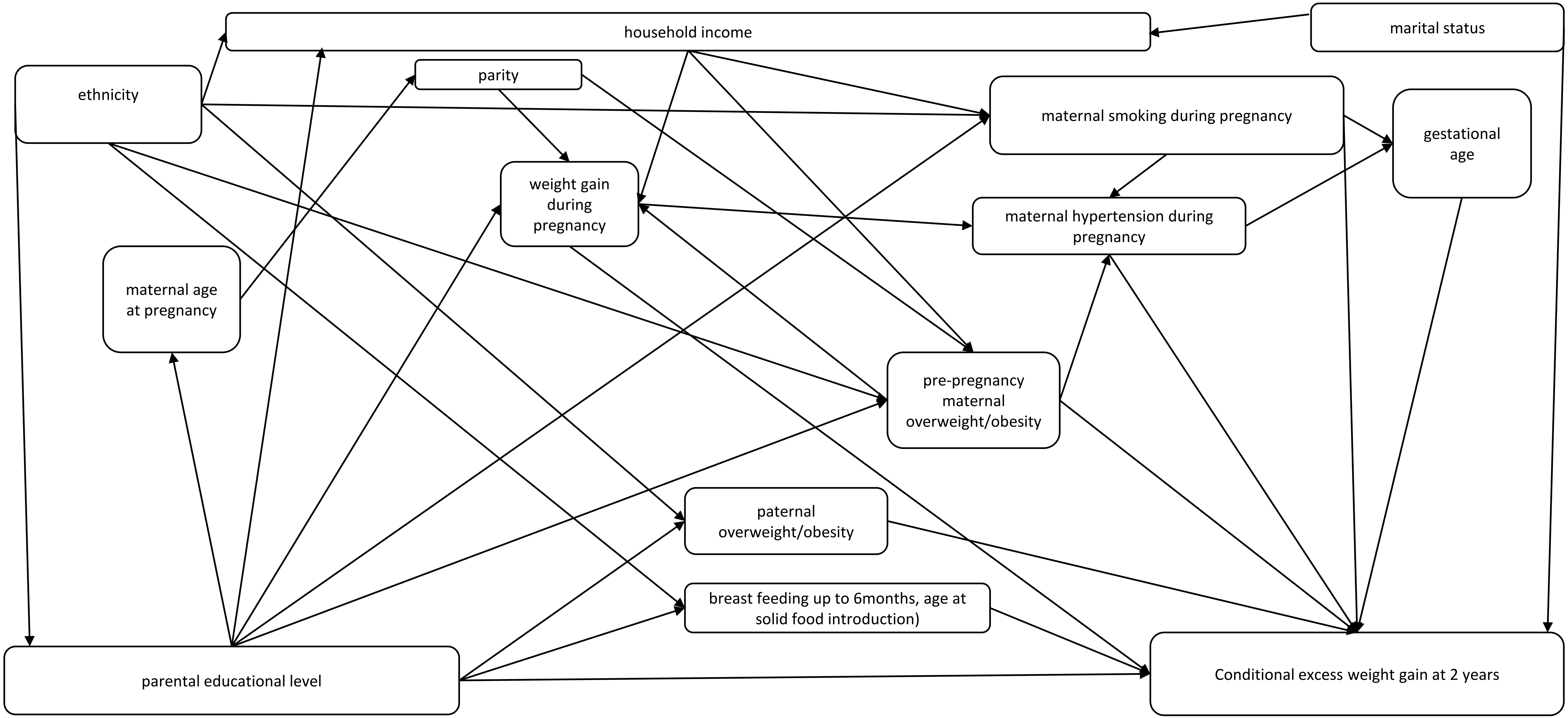

Supplementary figure S1 (a): causal diagram depicting the links between parental educational level and conditional excess weight gain at 2 years

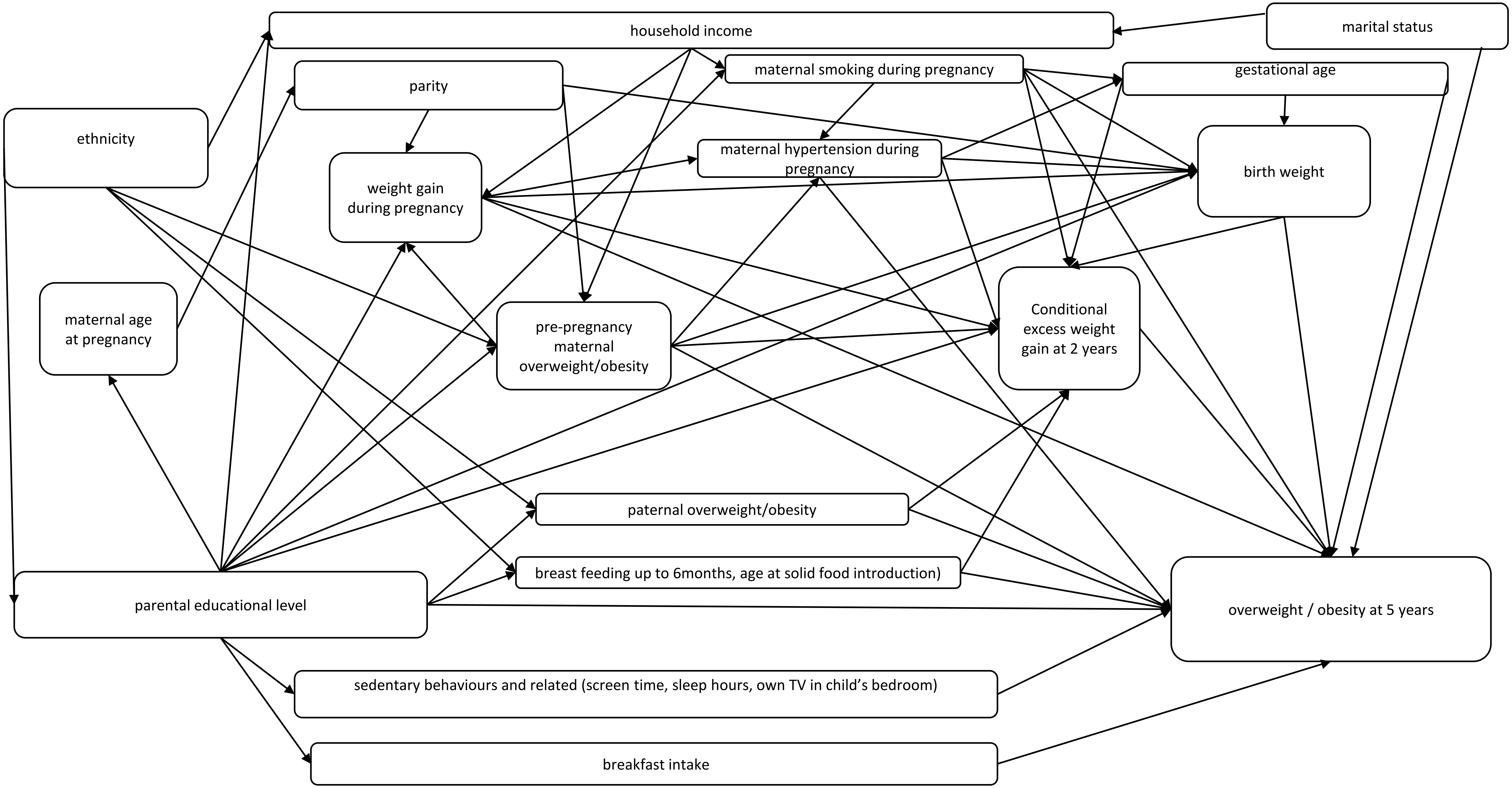

Supplementary figure S1 (b): causal diagram depicting the links between parental educational level and overweight/obesity at 5 years

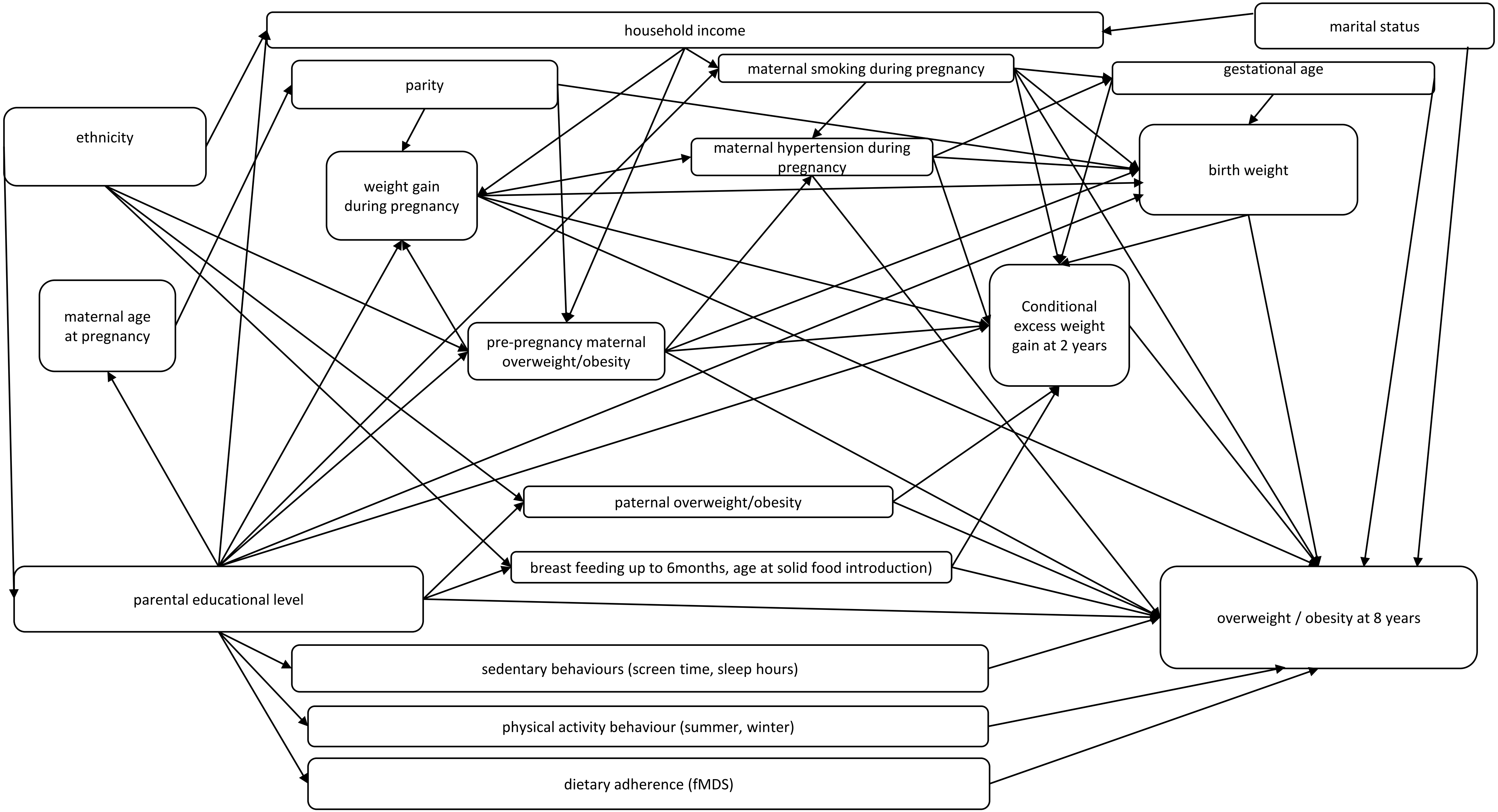

Supplementary figure S1 (c): causal diagram depicting the links between parental educational level and overweight/obesity at 8 years

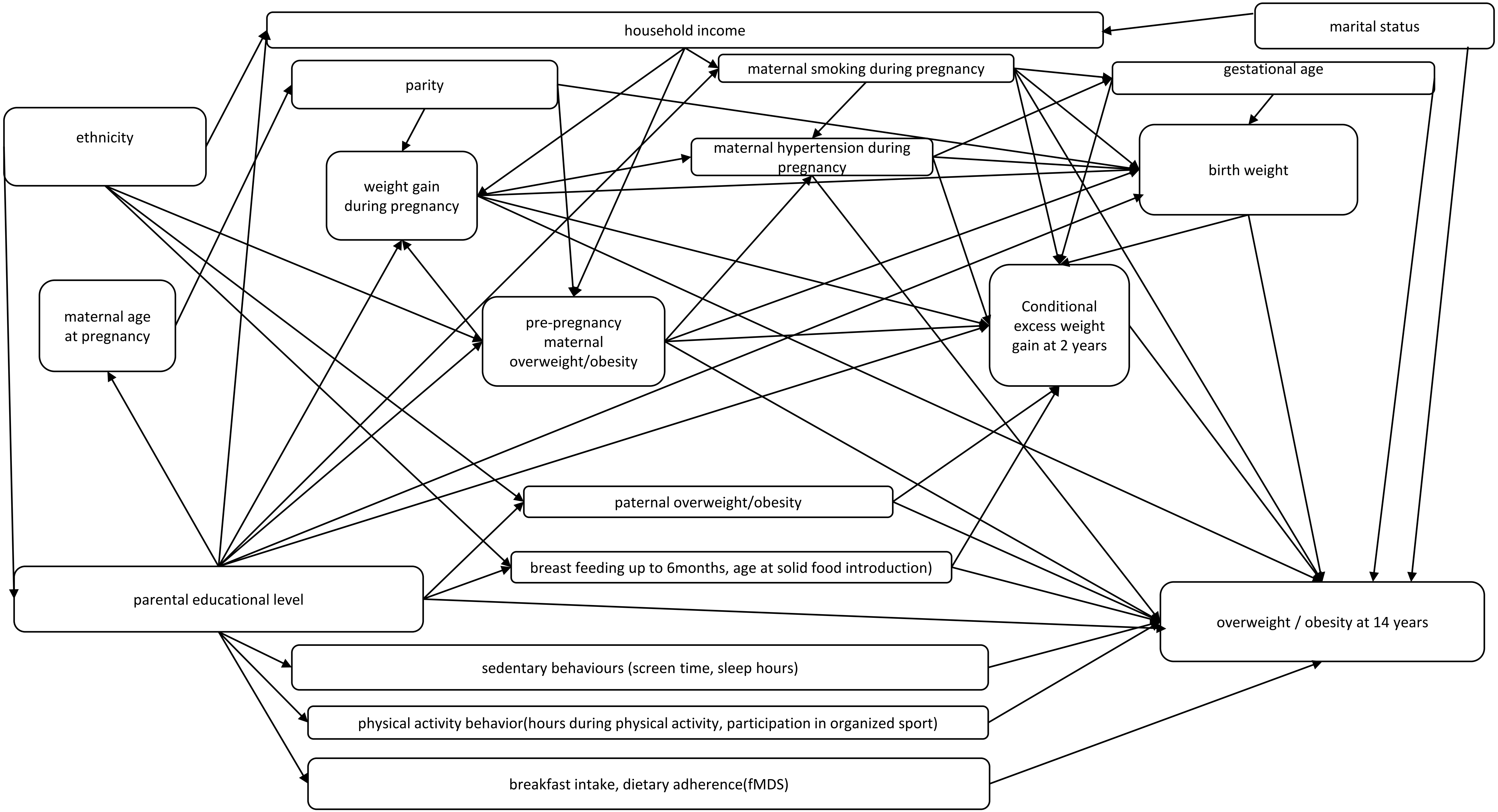

Supplementary figure S1 (d): causal diagram depicting the links between parental educational level and overweight/obesity at 14 years

Supplementary Table S1: mediating roles of prenatal factors (up to birth), early postnatal factors (up to 2 years) and child energy balance-related behaviours for the association between parental education and OW/OB at 5, 8, and 14 years, the MoBa Study

**Mediators included: Mediators included: prenatal factors (up to birth)**						
	5 years (n=27 134)		8 years (n=28 285)		14 years (n=11 278)	
	Overweight/obesity		Overweight/obesity		Overweight/obesity	
Parental Educational Level	**RR**	**95% CI**	**RR**	**95% CI**	**RR**	**95% CI**
Total Effect	1.23	1.16-1.29	1.55	1.41-1.71	1.45	1.27-1.66
Natural Direct Effect	1.07	1.01-1.14	1.20	1.08-1.33	1.11	0.97-1.28
Natural Indirect Effect	1.14	1.12-1.18	1.29	1.24-1.35	1.30	1.24-1.37
Proportion Mediated (%)	68.1		63.5		75.2	
**Mediators included: early postnatal factors birth to 2 years**						
	**RR**	**95% CI**	**RR**	**95% CI**	**RR**	**95% CI**
Total Effect	1.23	1.16-1.29	1.55	1.42-1.70	1.45	1.29-1.63
Natural Direct Effect	1.20	1.14-1.26	1.44	1.33-1.57	1.41	1.26-1.57
Natural Indirect Effect	1.03	1.001-1.05	1.07	1.04-1.11	1.03	1.003-1.06
Proportion Mediated (%)	15.3		18.6		9.4%	
**Mediators included: energy-balance-related behaviours**						
Total Effect	1.23	1.17-1.29	1.55	1.39-1.72	1.45	1.28-1.64
Natural Direct Effect	1.21	1.14-1.28	1.46	1.31-1.63	1.29	1.14-1.47
Natural Indirect Effect	1.02	1.01-1.03	1.06	1.04-1.07	1.12	1.09-1.15
Proportion Mediated (%)	10.3%		16.0%		34.8%	

All the models were adjusted for ethnicity, maternal civil status, and gestational age.

Sample Stata code for mediation analysis using inverse odds weighting

use "-",clear

NOTE: the highlighted(in bold colour) are variables names, lists, or parameters to be chosen by the user.

*define a user-written program;

capture program drop **IOWMICE2**

program **IOWMICE2**, rclass

preserve

* We requested 30 imputed datasets for the variables with missing data using Multiple Imputation via Chained Equations (MICE)

ice **outcome variable and variables with missing to be imputed**, saving(" _________ ",replace) m(30)

**use the imputed dataset

use " ___________", clear

*Retain estimates of predicted probability, inverse odds, and inverse odds weights for later use ;

capture drop **logodds2 predprob2 inverseodds2 wt_iow2**

*Insert regression of exposure variable(i.e. parental education) on mediators(m1, m2,m3) and covariates(c1,c2,c3). Mim command analyzes multiply imputed data. Storebv command stores regression results for later use;

mim, storebv: logit **E m1 m2 m3 c1 c2 c3**

*E stands for exposure variable; m stands for mediators , C stands for covariates

*Calculate predicted log odds and use that to calculate predicted probabilities and inverse odds;

predict **logodds2**, xb

gen **predprob2** = exp(**logodds2**)/(1+exp(**logodds2)**)

gen **inverseodds2** = ((1-**predprob2**)/**predprob2**)

*Calculate inverse odds weights;parental educational status is our exposure variable with a category of low and high education

gen **wt_iow2** = 1 if **peduc_cat2**==0

replace **wt_iow2 = inverseodds2** if **peduc_cat2**==1

* Insert the total effect regression here and retain estimate of total effects for later use ;

mim, storebv: glm **outcome E c1 c2**, fam(**poisson**) link(**log**) vce(**robust**)

matrix **bb_total2**= e(b)

scalar **b_total2=bb_total2** [1,2]

return scalar **b_total2=bb_total2** [1,2]

* Insert the direct effect regression here and retain estimate of direct effects. Calculate indirect effects as the difference between total effects and direct effects;

mim, storebv: glm **outcome E c1 c2** [pweight=**wt_iow2**], fam(**poisson**) link(**log**) vce(**robust**)

matrix **bb_direct2** = e(b)

scalar **b_direct2** = **bb_direct2**[1,2]

return scalar **b_direct2** = **bb_direct2**[1,2]

return scalar **b_indirect2** = **b_total2-b_direct2**

end

*Request bootstrapped estimates of indirect, direct and total effects. Provide the initial value of the random-number seed so estimates can be replicated later. Request 200 bootstrap replications ;

bootstrap r(**b_indirect2**) r(**b_direct2**) r(**b_total2**),reps(**200**)seed(**2122**): **IOWMICE2**

*********************************end***************************
